# Measurement of Platelet Function in an Experimental Stroke Model With Aspirin and Clopidogrel Treatment

**DOI:** 10.3389/fneur.2020.00085

**Published:** 2020-02-11

**Authors:** Franziska Lieschke, Yi Zheng, Jan Hendrik Schaefer, Klaus van Leyen, Christian Foerch

**Affiliations:** ^1^Neuroprotection Research Laboratory, Department of Radiology and Neurology, Massachusetts General Hospital, Harvard Medical School, Charlestown, MA, United States; ^2^Department of Neurology, University Hospital Frankfurt, Goethe-University, Frankfurt am Main, Germany

**Keywords:** dual antiplatelet therapy, aspirin, clopidogrel, flow cytometry, CD62P, CD41, hemorrhagic transformation, MCAO

## Abstract

Dual antiplatelet treatment (DAPT) increases the risk of tPA-associated hemorrhagic transformation (HT) in ischemic stroke. To investigate the effects of DAPT in rodents, reliable indicators of platelet function utilizing a minimally invasive procedure are required. We here established a fluorescence-based assay to monitor DAPT efficiency in a mouse model of ischemic stroke with HT. Male C57/BL6 mice were fed with aspirin and clopidogrel (ASA+CPG). Venous blood was collected, stimulated with thrombin, labeled with anti-CD41-FITC and anti-CD62P-PE, and analyzed by flow cytometry. Subsequently, animals were subjected to experimental stroke and tail bleeding tests. HT was quantified using NIH ImageJ software. In ASA+CPG mice, the platelet activation marker CD62P was reduced by 40.6 ± 4.2% (*p* < 0.0001) compared to controls. *In vitro* platelet function correlated inversely with tail bleeding tests (*r* = −0.8, *p* = 0.0033, *n* = 12). Twenty-four hours after drug withdrawal, platelet activation rates in ASA+CPG mice were still reduced by 20.2 ± 4.1% (*p* = 0.0026) compared to controls, while tail bleeding volumes were increased by 4.0 ± 1.4 μl (*p* = 0.004). Conventional tests using light transmission aggregometry require large amounts of blood and thus cannot be used in experimental stroke studies. In contrast, flow cytometry is a highly sensitive method that utilizes small volumes and can easily be incorporated into the experimental stroke workflow. Our test can be used to monitor the inhibitory effects of DAPT in mice. Reduced platelet activation is indicative of an increased risk for tPA-associated cerebral hemorrhage following experimental stroke. The test can be applied to individual animals and implemented flexibly prior and subsequent to experimental stroke.

## Introduction

The use of dual antiplatelet treatment (DAPT) in the acute phase of stroke is becoming increasingly common (1, 2). Apart from patients prescribed DAPT for other indications (3), this includes the application of DAPT in patients with transient ischemic attacks and minor strokes, as well as DAPT treatment during acute revascularization procedures (4, 5). By irreversibly impairing platelet function, DAPT increases the risk of hemorrhagic complications after thrombolysis with tPA (6–8).

Current European and American guidelines do not recommend to perform platelet function tests during the acute phase of ischemic stroke (9, 10). Although platelet function testing has become widely accessible with the use of point of care devices, investigations whether patients could potentially benefit are still lacking. On the other hand, rapid testing of the platelet count is a well-established standard in the treatment of stroke. Here, low platelet counts are excluded from tPA treatment due to the significantly increased risk of hemorrhagic transformation (HT) in platelet depleted patients (11). However, the question of whether and how platelet function contributes to tPA-associated HT has not been answered to date.

To address this gap of knowledge, we recently established a model of tPA-associated hemorrhagic transformation (HT) in mice pretreated with Aspirin and Clopidogrel [ASA+CPG, (12)]. While establishing antiplatelets in rodents, the individual hemostatic status of the single mouse becomes relevant. We therefore looked for a simple platelet function test to check for individual drug response.

Conventional platelet function tests like light transmission aggregometry (LTA) require significant volumes of blood (13), which means either collecting a lethal amount of blood from one mouse or collecting and pooling blood from different animals (14). As a result LTA is too invasive to be used as a measure of platelet status at the time of experimental stroke surgery. A simple-to-conduct but unspecific method is the tail bleeding test, which illustrates platelet adhesion *in vivo* and corresponds to the bleeding time test formerly used in patients (15, 16). The principle underlying this test is that platelet function directly affects primary hemostasis after tissue damage, resulting in prolonged bleeding time (time to complete cessation of the bleeding) and increased blood volumes during testing in mice. It can only be done once at the end of an experiment, and precludes the evaluation of drug efficacy in advance and repeated measurements. Accordingly, methods are required that utilize small sample volumes. Flow cytometry is a highly sensitive method requiring only small volumes of blood. Platelet activation assays based on flow cytometry have been introduced (17–19) but at present they are not widely used and have not been tested in the context of experimental stroke studies.

The aim of this study was to demonstrate the feasibility of incorporating flow cytometry-based platelet function testing in a mouse model of DAPT followed by an ischemic stroke and tPA infusion. In addition to monitoring the efficacy of antiplatelet therapy, we also sought to further investigate the impact of platelet function on the development of HT in order to predict bleeding risks and to define inclusion and exclusion criteria for subsequent experiments.

## Materials and Methods

### Animals and Experimental Design

**Figure 1:**
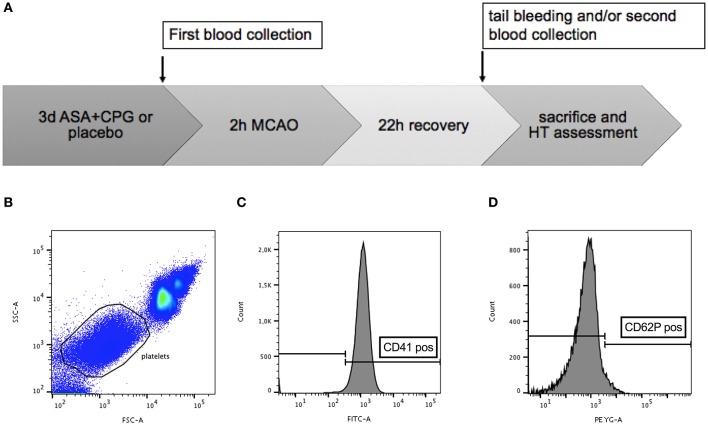
**(A)** Timeline diagram of the experimental procedures: platelet function in control and ASA+CPG mice was tested *in vitro* using flow cytometry at the end of 3 days pretreatment. Mice were subjected to 2 h MCAO and tPA administration. Twenty-two hours later, tail bleeding tests and a second flow cytometry analysis were performed, followed by sacrifice and histological assessment of hemorrhage. **(B–D)** Gating strategy: Ungated whole blood **(B)**. Gated cells scored high for platelet identification marker FITC-CD41 **(C)** and low for the platelet activation marker PE-CD62P **(D)**, consistent with the properties of resting platelets (Analysis performed with FlowJo).

In total 28 male C57/B6 mice (Jackson, Bar Harbor, ME, USA) aged 9–10 weeks with a mean body weight of 26 ± 2g were used in this study. All experiments conformed to an institutionally approved protocol in accordance with the National Institute of Health's guide for the care and use of laboratory animals. We used exclusively male animals to limit variability due to sex differences. The operators performing surgical procedures and the investigators evaluating data were blinded to the treatment groups. At first, we assessed platelet function *in vitro* using our flow cytometry-based assay (as explained below) in mice treated with ASA+CPG and in controls. Mice were then subjected to 2 h MCAO followed by tPA administration. Twenty-four hours later, standard tail bleeding tests were conducted (14, 20), and mice were sacrificed to quantify HT development. A second *in vitro* analysis of platelet function was performed at the end of the experiment in order to demonstrate the feasibility of monitoring treatment effects beyond drug withdrawal (Figure 1A).

### Antiplatelet Pretreatment

Mice were allocated randomly to a treatment or control group. ASA (Bayer Health Care, Morristown, NJ, USA) and CPG (Dr. Reddy's Laboratories Ltd., Beverley, UK) diluted in drinking water (ASA 0.4 mg/mL, CPG 0.15 mg/mL) were supplied *ad libitum* for 72 h. A water consumption of 15 ml/100 g per 24 h was assumed, providing an estimated daily intake of 60 mg/kg ASA and 22.5 mg/kg CPG per mouse. These dosages were selected based on previous experimental stroke studies (14, 21). Control mice received regular drinking water.

### Flow Cytometry Based Platelet Function Test

*In vitro* platelet function testing was based on the platelet expression of CD41 and CD62P. We used the platelet marker CD41 to distinguish platelets from other events. CD62P is usually located in the membrane of platelet α-granules in the cytoplasm and only translocates to the plasma membrane after platelet activation (22). By detecting the surface co-expression of these antigens following thrombin treatment using a BD LSRII analyzer (Becton-Dickinson, San Jose, CA, USA), we gained an individual platelet activation rate for each single mouse. A high activation rate (indicated by increased CD62P expression) demonstrates sufficient platelet function, whereas a lower activation rate indicates impaired platelet function due to the partial inhibition of platelets, hence also serving as an indicator for an efficient anti-platelet treatment. Briefly, mice were anesthetized with isoflurane (1.25–1.5% in a nitrous oxide/oxygen mixture with spontaneous respiration). Venous blood (10–20 μl) was collected from the left jugular vein and given into sodium citrate (final 0.32%, Sigma) to prevent unintended platelet activation and blood clotting. Five microliters of whole blood were diluted in thirty microliters of PBS containing 0.32% sodium citrate. Fifteen microliters of vehicle or agonist solution were added, where the vehicle was composed of PBS containing 0.32% sodium citrate, and the agonist solution consisted of thrombin (final 2 μ/ml) supplemented with 10 mM GPRP (final 2.5 mM) and 6 mM CaCl2. The samples were then incubated shaking at 37°C for 5 min. Mouse blood was incubated with 1:100 dilution of CD41-FITC and CD62P-PE monoclonal antibodies (BD Biosciences and eBioscience™, respectively) for 15 min at room temperature in the dark. After staining, samples were fixed with 650 μl of fixative solution containing 0.1% formalin, 0.1% dextrose, and 0.2% BSA in PBS. Labeled, diluted and fixed samples were analyzed. Platelets were gated based on their characteristic forward and side scatter properties (Figure 1B), as well as for antiplatelet immunoreactivity for CD41 (Figure 1C). The third gate was set on a positive staining with monoclonal antibody CD62P-PE (Figure 1D). Analyses were performed with BD FACS DIVA software (BD Biosciences) and FloJo (v10).

### MCAO

Animals were anesthetized, and a 6–0 silicone-coated monofilament was introduced into the right internal carotid artery until the tip occluded the ostium of the MCA. Regional cerebral blood flow was monitored by laser doppler flowmetry with the use of a probe fixed to the intact skull above the territory of the right MCA. Rectal temperature was maintained between 36.5 and 37°C with a heating pad. After surgery, animals were allowed to recover from anesthesia. Hundred and twenty minutes after MCAO, the filament was withdrawn to initiate reperfusion, and 62.5 μL of tPA (4 mg/ml, final 10 mg/kg BW) were given by intravenous infusion into the right jugular vein over 15 min using a perfusion pump. 0.1 mg/kg BW buprenorphine hydrochloride (Buprenex®, Reckitt Benckiser Healthcare Ltd, UK) were administered at the end of all procedures.

### Standard Tail Bleeding

To control for confounders, we first assessed the body temperature: Hypothermic mice were excluded, while normothermic mice were anesthetized and placed on a heating pad in prone position. A distal 5 mm segment of the tail was amputated with a razor blade. Tails were immediately inserted into microtubes containing 1 microliter saline placed in a water bath pre-warmed to 37°C. After 3 min, the tail was removed from the microtube, and hemostatic measures were taken by electro-cauterization using Bovie®. The microtube was vortexed, and ultrasound was applied for 15 s to lyse erythrocyte cell membranes. 3 × 100 μL were transferred to a 96 well plate containing 40 μL of Drabkin's reagent and incubated for 15 min. Absorption rates at 540 nm were determined using a SpectraMax M5 photometer. Bleeding volumes were calculated using a standard curve (14, 20, 23).

### HT Determination

For HT measurement in the brain, mice were lethally anesthetized and perfused transcardially with saline. The brains were removed and sectioned into 1 mm thick slices and photographed. HT was assessed as red areas in brain sections, outlined and measured using ImageJ. Hemorrhages were classified according to the ECASS II morphologic definitions (24, 25) adapted to animal models as used in previous publications (12, 26, 27). Therefore, every section was individually scored on a 5 point ordinal scale (1 = no HT; 2 = hemorrhagic infarction type 1; 3 = hemorrhagic infarction type 2; 4 = parenchymal hemorrhage type 1; 5 = parenchymal hemorrhage type 2) and an overall grade for every brain was determined according to the highest grade occurring among the sections.

### Exclusion Criteria

Death within the 22 h recovery period led to exclusion from further assessment. Post-stroke tail bleeding tests were not performed in surviving animals when the general condition was too severe (indicated by a low body temperature (<32°C) and slow breathing rate, for details please see Supplementary Material).

### Post-stroke Flow Cytometry Analysis of Platelet Function

Post-stroke flow cytometry analysis was performed with blood collected from 5 randomly chosen control mice and 3 ASA+CPG treated mice by cardiac puncture at the time point of sacrifice and conducted as described above.

### Statistics and Data Analysis

Data is presented as mean ± SEM. Values were tested for Gaussian distribution with D'Agostino-Pearson omnibus normality test and Kolmogorov test. Statistical significance was determined for two group comparisons with Welch's *t*-test or Mann-Whitney test, and data sets were considered different if *p* < 0.05. Correlations were calculated with Spearman's test. All statistics were performed using Prism 7 graphpad software.

## Results

### Pre-stroke Platelet Function Testing

**Figure 2:**
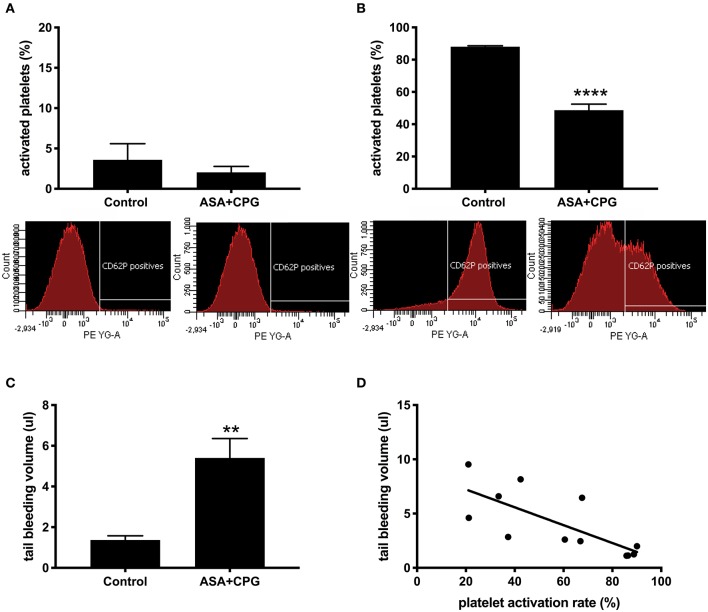
**(A)** Platelet activation rates in unstimulated samples: Control: 3.6 ± 2.0%, *n* = 12; ASA+CPG: 2.0 ± 0.7%, *n* = 15, *p* = 0.3649 with selected example images of fluorescence intensity for platelet activation marker anti-CD62P-PE in unstimulated blood samples (FACSDiva software). **(B)** Platelet activation rates in thrombin stimulated samples: Controls: 88.0 ± 0.6%, *n* = 12; ASA+CPG: 47.5 ± 3.8%, *n* = 15, *p* < 0.0001, images of anti-CD62P-PE fluorescence intensity after *in vitro* thrombin stimulation. **(C)** Blood volumes in post-stroke tail bleeding testing: Controls: 1.4 ± 0.2 μl/3 min, *n* = 4; ASA+CPG: 5.4 ± 0.9 μl/3 min, *n* = 8, *p* = 0.004. **(D)** Correlation of *in vitro* platelet function with *in vivo* tail bleeding volumes: Linear regression: *R*^2^=0.5518, y = −0.08175 (95% CI: −0.134 to −0.03)*X+8.846). Correlation: *r* = −0.7902, *p* = 0.0033, *n* = 12. **p < 0.01; ****p < 0.0001.

In control mice (*n* = 12) platelet pre-activation in unstimulated samples was 3.6 ± 2.0% vs. 2.0 ± 0.7% in unstimulated blood obtained from ASA+CPG treated mice (*n* = 15, *p* = 0.365, Figure 2A). *In vitro* stimulation with thrombin platelet activation was 88.0 ± 0.6% in controls compared to 47.5 ± 3.7% in ASA+CPG treated mice (*p* < 0.0001, Figure 2B), indicating a partial platelet inhibition in these animals. Thus, we sufficiently demonstrated the *in vitro* detection of ASA+CPG drug effects in whole blood collected from mice following 3 days of oral drug administration.

### Implementation Into Experimental Stroke

Subsequently, we subjected all mice to 2 h MCAO and tPA infusion, followed by a 22 h recovery period before performing tail bleeding tests (Figure 1A). During the recovery period, 4 control mice and 2 ASA+CPG treated mice died. We examined the deceased animals, focusing on intracerebral- or gastrointestinal bleeding or any other obvious hemorrhagic complication. Neither the ASA+CPG mice nor the control mice showed severe hemorrhages, suggesting that mortality was mainly related to the fairly severe ischemia induced by 2 h of MCAO. Tail bleeding volumes measured in 8 ASA+CPG treated mice were on average 5.4 ± 0.9 μl compared to 1.4 ± 0.2 μl measured in 4 control mice (*p* = 0.004, Figure 2C), suggesting platelet inhibition was still effective 24 h after cessation of treatment.

In comparing the individual results of pre-stroke platelet function testing with post-stroke tail bleeding volumes (the standard *in vivo* measure of platelet function), we found that low platelet activation rates upon *in vitro* thrombin stimulation correlated significantly with increased blood loss in the tail bleeding test and vice versa (*r* = −0.7902, *p* = 0.0033, *n* = 12; Figure 2D), suggesting our method is feasible to depict platelet function in mice.

**Figure 3:**
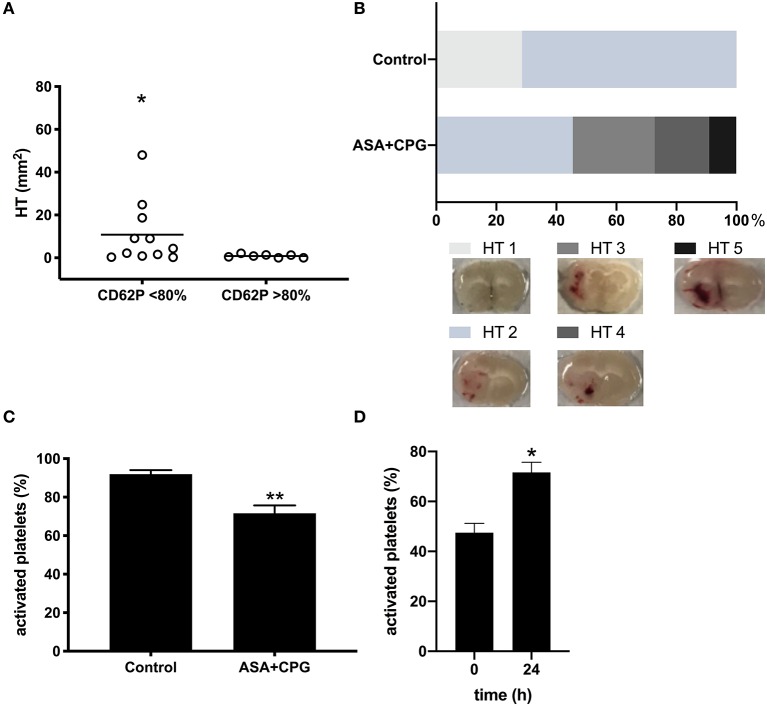
**(A)** Hemorrhage area measurements in brain sections: Platelet activation rate >80% CD62P expression: 0.9 ± 0.3 mm^2^, *n* = 7; Platelet activation rate <80% CD62P expression: 10.8 ± 4.5 mm^2^, *n* = 11, *p* = 0.0335. **(B)** Morphological classification of the bleeding types: 0/7 control mice were rated HI-2, compared to 6/11 mice in the ASA+CPG (*p* = 0.0125). **(C)** Platelet activation rates in thrombin stimulated samples (post-stroke): Controls: 91.9 ± 2.1%, *n* = 5; ASA+CPG: 71.6 ± 4.1%, *n* = 3, *p* = 0.0026. **(D)** Platelet activation following thrombin stimulation pre- and post-stroke in ASA+CPG pretreated mice: Platelet activation rates increased significantly by 22.9 ± 8.9% (*p* = 0.0204) within 24 h between the two measurements. *p < 0.05; **p < 0.01.

Investigating the effect of platelet function on HT development, we found that HT did not occur in mice that demonstrated beforehand high activation rates upon *in vitro* stimulation with thrombin (CD62P expression >80%), whereas mice showing reduced platelet activation rates (CD62P expression <80%) were at higher risk for HT development (CD62P expression >80%: 0.9 ± 0.3 mm^2^; CD62P expression <80%: 10.8 ± 4.4mm^2^, *p* = 0.0335, Figure 3A). No hemorrhages were found remote from the infarct. The morphological classification of the HT types showed that 0/7 control mice were rated HI-2, compared to 6/11 mice in the ASA+CPG (*p* = 0.0125, Figure 3B, Supplementary Table I).

### Post-stroke Platelet Function Testing

In analogy to the post-stroke tail bleeding testing, we performed a second *in vitro* platelet function testing post-stroke in randomly selected mice at the time point of sacrifice. In ASA+CPG pretreated mice, platelet activation upon *in-vitro* thrombin stimulation 24 h after drug withdrawal was 71.6 ± 4.1% compared to 91.9 ± 2.1% in controls (Figure 3C, *p* = 0.0026). Although the antiplatelet effect of ASA+CPG pretreatment was still detectable 24 h after stroke and drug withdrawal, platelet function in ASA+CPG pretreated mice already improved significantly compared to the platelet activation rates of the first *in vitro* testing (Figure 3D platelet activation rates in ASA+CPG mice pre-stroke 47.5 ± 3.8% vs. 71.6 ± 4.1% post-stroke). We here present the feasibility of repeated sampling within a stroke model using our flow cytometry-based platelet function assay.

## Discussion

With this study, we successfully developed a practical and straightforward method to test platelet function *in vitro* using flow cytometry, and we applied this assay to ASA+CPG treated mice in the context of experimental stroke studies in order to monitor antithrombotic treatment efficacy. Furthermore, we demonstrated that our method can be implemented at different time points within the stroke model and that repeated measurements are also feasible. Our method can be used to identify animals that are at a higher risk for developing HT, in order to study the pathophysiology and to explore new therapeutic approaches.

Thus, far, tail bleeding has been the only measure that assesses platelet function in an individual mouse without the need to pool blood from different subjects (which is needed for LTA). However, when it comes to its implementation into stroke models, this approach faces serious limitations. First, tail bleeding testing can only be performed at the end of an experiment before sacrifice due to a high risk of severe and uncontrolled bleeding. Secondly, the tail bleeding test is relatively unspecific (20). It can be affected by secondary hemostasis, blood pressure and body temperature. Another disadvantage is that mice cannot be tested repetitively, precluding dynamic measurements. In contrast, our flow cytometry based technique of platelet function analysis requires only 0.5–1% of the total blood volume of a mouse, allowing for repeated sampling.

We showed that there was no significant difference in platelet activation in unstimulated blood between ASA+CPG treated mice and control mice prior to stroke, which indicates that our samples were handled equally, resulting in acceptable pre-activation rates. However, pre-activation rates in ASA+CPG treated mice were slightly reduced compared to controls, which is in line with the findings of Kassassir et al. (19), who demonstrated that pre-activation rates in mice intravenously treated with cangrelor (another ADP-receptor antagonist like CPG) were reduced compared to untreated controls (19).

As we had hypothesized, ASA+CPG treatment led to significantly reduced platelet activation rates upon *in vitro* stimulation with thrombin. These treatment effects occurred in a comparable range to previously reported results in humans (28), although relatively high treatment dosages (of 60 mg/kg ASA and 22.5 mg/kg CPG) were used. The faster metabolism of mice and different administration protocols (crushing and diluting tablets which are designed for enteric resorption in humans) may explain these comparable effects in spite of a higher absolute dose (19, 22, 29, 30). Ultimately, while there was some variability in the response, we did not identify any non-responders, and all ASA+CPG mice showed reduced platelet reactivity.

Accordingly, we demonstrated that the impaired platelet function after ASA+CPG pre-treatment is strongly correlated with higher tail bleeding volumes. Thus, our method can be used to identify mice with decreased platelet function. Based on this, cohorts with an overall increased risk of HT could be identified. However, HT prediction on an individual animal level remains difficult. Similar to humans, not every pretreated mouse developed HT following thrombolytic treatment, although mice that showed hemorrhages generally had lower activation rates. These findings are in line with previous attempts to better stratify the risk of tPA-associated HT in human patients (7, 31–36), which aimed to define risk scores and predictors for HT. With only modest predictive power, those models failed to identify common key contributors. More research on pre-stroke platelet function and its impact on later HT development and neurological outcome may help to fill this gap of knowledge.

The current literature also revealed that despite the higher risk of tPA-associated HT in patients on DAPT, mortality and neurologic outcomes did not differ from patients without DAPT; thus, the benefit of tPA seemed to outweigh its risk (37–39). In order to get a clearer picture, it may be useful to implement platelet function testing during the early phase of acute ischemic stroke through using flow cytometry-based assays or point of care devices, which have already replaced the previous standard screening methods for platelet dysfunction (40). Testing the platelet function rather than the platelet count could potentially make discussion about platelet threshold superfluous (41).

We also need to address the limitations of our study. In conventional aggregation assays, typically ADP is used to demonstrate CPG effects (32). ADP receptor activation in mice leads to the release of platelet dense granules but not to the release of alpha granules (14). We used CD62P which is only located in alpha granules; thus, ADP stimulation could not be illustrated using anti-CD62P. Another consideration addressing the flow cytometry test relates to our method of quantification. An accepted strategy to quantify platelet aggregation using flow cytometry is the determination of the loss of single platelets due to aggregation upon platelet activation (17, 18, 28, 42, 43). Since we were using two separate samples when comparing unstimulated vs. stimulated blood, we were unable to consistently detect and quantify the loss of single platelets in these samples. A complementary strategy to deal with these limitations for future studies is to use only one sample and repeat measurements before and after platelet stimulation. However, this would require the platelet count to be determined. Which implies another limitation to our study, since platelet function can be affected by platelet count (44, 45). Also, we have not tested platelet function following long-term treatment yet, in which other hemostatic factors besides platelets may also be affected. In addition, our study was designed as a single-center study at the Neuroprotection Research Laboratory (Massachusetts General Hospital), and needs to be confirmed by replication in other laboratories. A last but major concern is the validation of our flow cytometry assay by using tail bleeding. Since platelet function *in vitro* and *in vivo* may differ (44, 46, 47), it is insufficient to base examinations on either *in vitro* or *in vivo* only. By combining the FACS analysis with tail bleeding, we intended to achieve a complementary insight into the hemostatic status of an individual mouse. As expected, tail bleeding volumes were increased in the pretreated mice 24 h after the ischemic event. At the same time, FACS analysis of blood from the same mice showed reduced platelet activation in the DAPT treated mice, and these results correlated well (*r* = −0.7902, *p* = 0.0033, Figure 2D). We also considered cross-validation by comparison with aggregometry, but decided against it because (a) Due to the large volume required for the aggregometer used in the MGH pathology core facility, we would have had to pool the blood of several mice, which practically could not be combined with a stroke model; (b) The procedure used to initiate platelet activation differs significantly from our approach. For example, the core facility uses ADP or arachidonic acid to activate the platelets *in vitro*, while we used thrombin in our study; (c) Measurements with aggregometers are manufacturer and laboratory specific, which implies a restricted comparability between centers. These limitations would have ruled out a quantitative comparison of the two methods. However, the previous study by Lauer et al. demonstrated that the dosages of ASA and CPG used in our study were sufficient to significantly reduce platelet aggregation as measured by aggregometry (14), which is in line with our current results based on FACS measurements.

In summary, we describe a versatile and easy-to-implement tool to investigate the impact of platelet function in experimental stroke. We demonstrated that ASA+CPG treatment resulted in significantly impaired platelet function contributing to an increased risk of HT after experimental stroke and tPA administration. Our test can be used in animal studies to monitor drug effects. To identify and exclude mice with insufficient platelet inhibition from future experiments, it can be implemented at various times within the model and repeated using dynamic measurements. We here offer an experimental model to better investigate and understand the mechanisms underlying the impaired platelet function contributing to HT.

## Data Availability Statement

All datasets generated for this study are included in the article/Supplementary Material.

## Ethics Statement

The animal study was reviewed and approved by Institutional Animal Care and Use Committee (IACUC) at Massachusetts General Hospital (MGH).

## Author Contributions

CF, KL, and FL conceived the idea, designed the model and analyzed the data. FL carried out the experiments and performed the flow cytometry measurements. YZ performed the MCAO surgery. FL processed the experimental data, performed the statistical analysis, drafted the article, and designed the figures. JS aided in interpreting the results. KL and CF supervised the work. CF and KL made critical revisions of the article for important intellectual content. All authors discussed the results and commented on the article.

### Conflict of Interest

The authors declare that the research was conducted in the absence of any commercial or financial relationships that could be construed as a potential conflict of interest.
