# Supplementary material for: Measurement of Platelet Function in an Experimental Stroke Model With Aspirin and Clopidogrel Treatment
Source: Front Neurol. 2020 Feb 11;11:85. doi: 10.3389/fneur.2020.00085 (PMC7026492; doi:10.3389/fneur.2020.00085)
Supplement: Supplementary file 1 [file Table_1.DOCX]

Supplementary Material

# Supplementary Data

## Sample size calculation

Power calculations were based on the normalized hemorrhage observed in a preliminary study comparing MCAO and tPA-treated mice with or without ASA+CPG pretreatment (Supplemental Figure 1). Using a power of 0.8 and a significance level of 0.05, an approximate sample size required of 6 animals per group was calculated. Anticipating a larger variability of HT within ASA+CPG pretreated animals (not every pretreated mouse consequently developed HT) and to compensate for dead or excluded animals, we randomized a larger number of mice per group. This preliminary study was also used for our recent mouse study on ASA+CPG in ischemic stroke^1^.

## Overall HT Types

Hemorrhages were classified according to the ECASS II morphologic definitions ^2,3^ adapted to animal models as used in previous publications ^1,4,5^. Therefore, every section was individually scored on a 5 point ordinal scale (class 1=no HT; 2=hemorrhagic infarction type 1; 3=hemorrhagic infarction type 2; 4=parenchymal hemorrhage type 1; 5=parenchymal hemorrhage type 2) and an overall grade for every brain was determined according to the highest grade occurring among the sections. The results are shown in the supplementary table I.

# Supplementary Figures and Tables

## Supplementary Figure 1


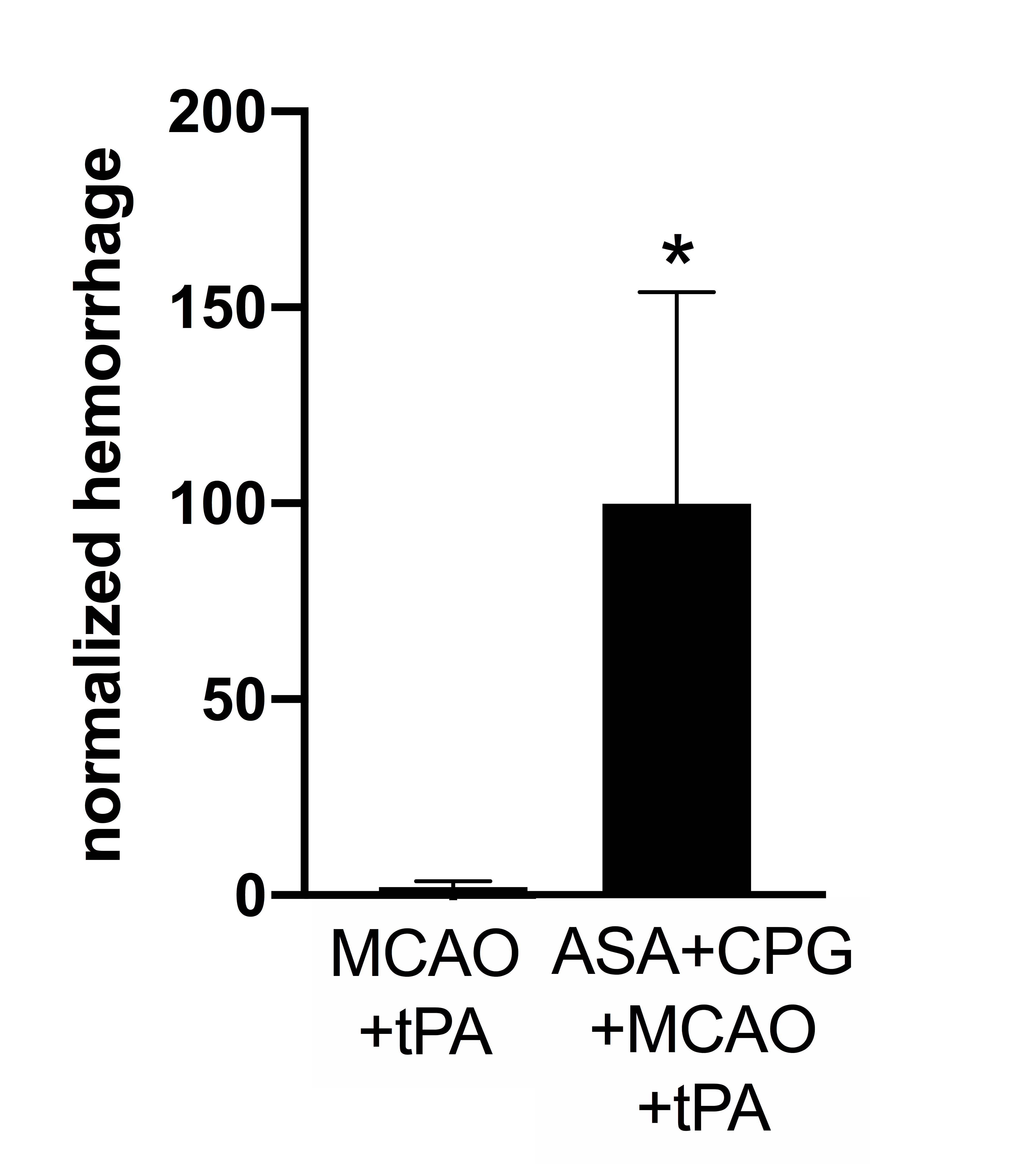


**Supplemental Figure 1**. Preliminary experiment on ASA+CPG mice vs. control mice. Control mice total hemorrhage (combined brain surface and sections measurements) was only 2.2±1.4% of the total hemorrhage determined in ASA+CPG mice.

## Supplementary Table I

| Overall HT types | HT grade 1 | HT grade 2 | HT grade 3 | HT grade 4 | HT grade 5 | Sum (mice total) |
| --- | --- | --- | --- | --- | --- | --- |
|  | 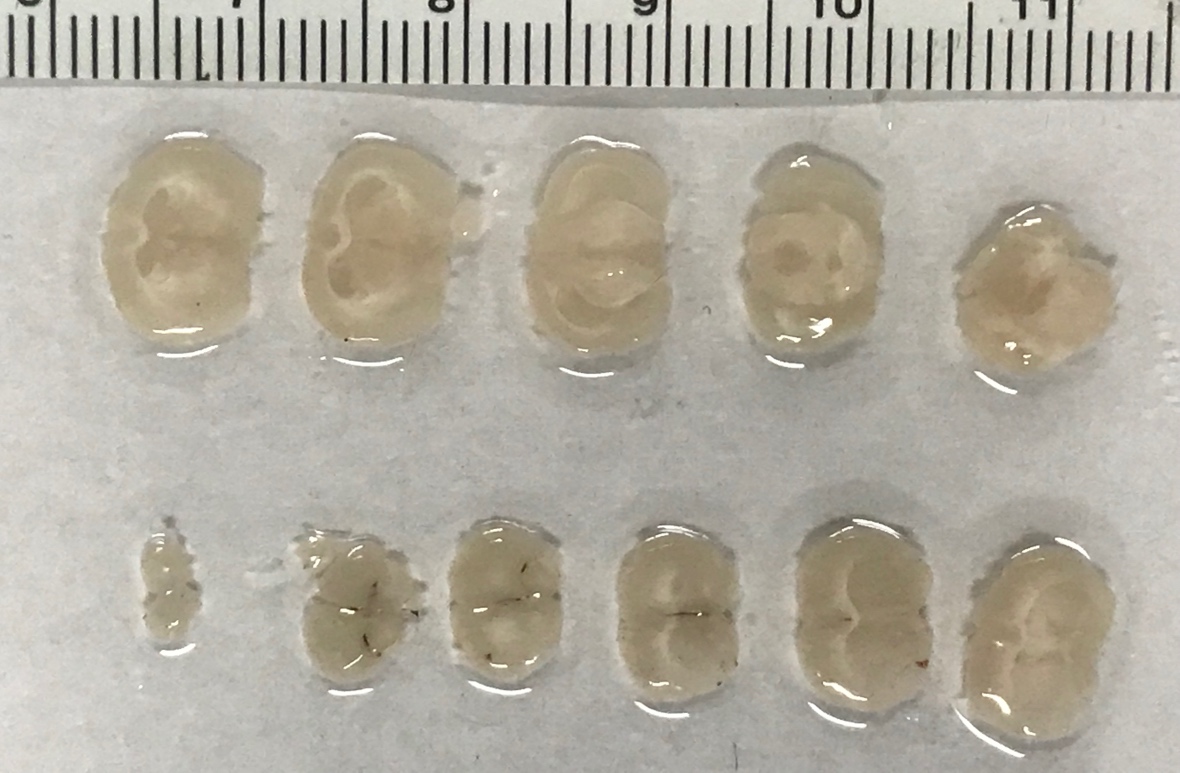  non hemorrhage | 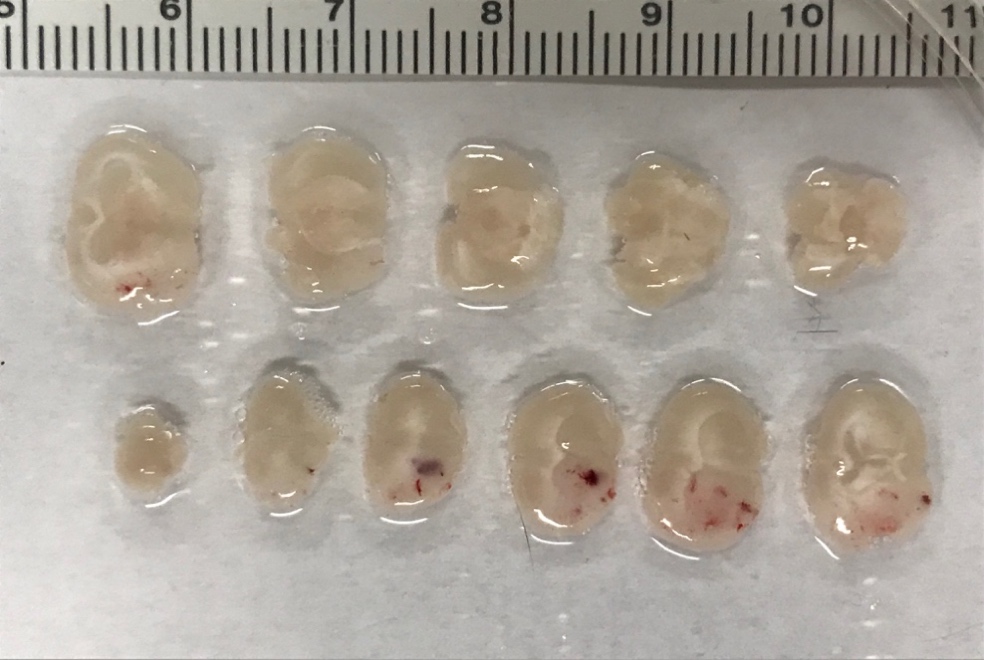  hemorrhagic infarction type 1 (HI-I): small petechiae along the boundary of the infarct | 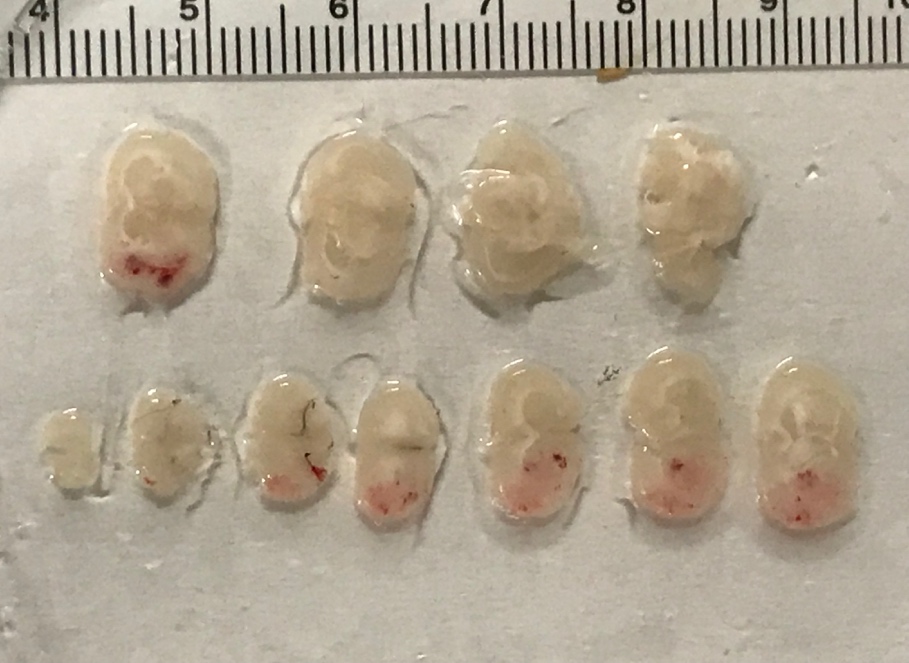  hemorrhagic infarction type 2 (HI-II): confluent petechiae within the infarct | 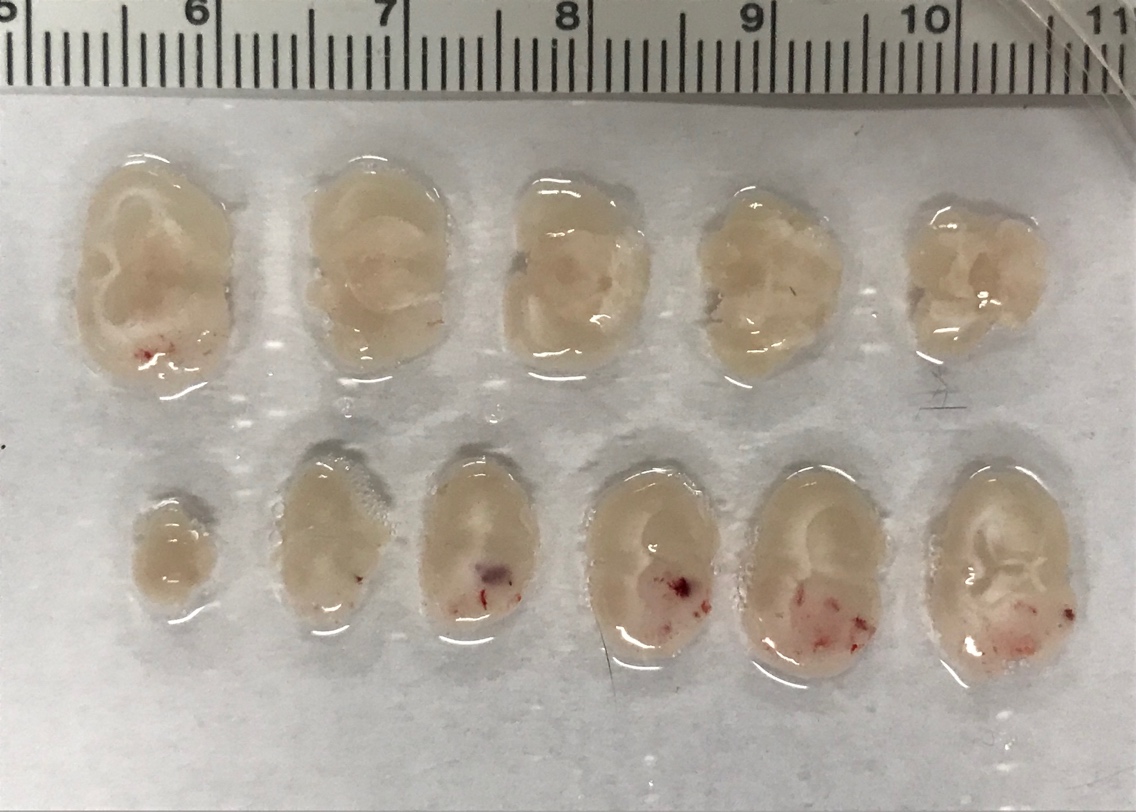  parenchymal hematoma type 1 (PH-I): blood clots within the infarct (<30% of the ischemic lesion size) | 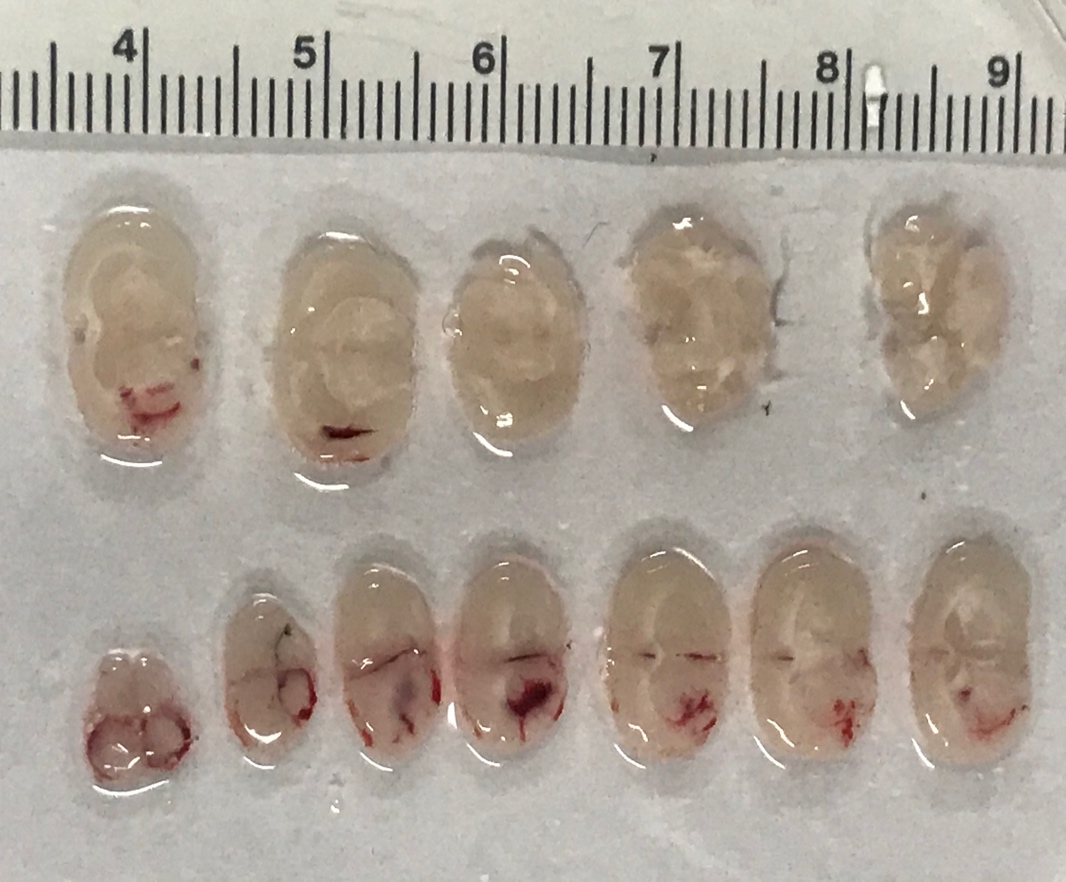  parenchymal hematoma type 1 (PH-II): clots in >30% of the infarct |  |
| ASA+CPG  +MCAO  +tPA | 0 | 5 | 3 | 2 | 1 | 11 |
| MCAO  +tPA | 2 | 5 | 0 | 0 | 0 | 7 |

**Supplementary Table I.** Morphological classification of the bleeding types. MCAO= transient middle cerebral artery occlusion; ASA+CPG= Aspirin and Clopidogrel; HT= Hemorrhagic Transformation; HI= Hemorrhagic infarction; PH= Parenchymal hematoma; tPA= tissue plasminogen activator.

## Supplementary Table II

| Experimental group | Included samples | Excluded samples (platelet count less than 1000 events-due to poor blood collection) | Excluded due to processing error  (failed labelling) | Total blood samples |
| --- | --- | --- | --- | --- |
| Control | 12 | 0 | 0 | 12 |
| ASA+CPG | 15 | 0 | 1 | 16 |

**Supplementary Table II.** Inclusion and exclusion criteria pre-stroke **flow cytometry** based platelet function testing.

## Supplementary Table III

| Experimental group | Included mice | Excluded mice- died during observation period | Excluded mice- poor physical condition (low body temp, slowly breathing) | Total mice |
| --- | --- | --- | --- | --- |
| MCAO+tPA | 4 | 4 | 4 | 12 |
| ASA+CPG+MCAO+tPA | 8 | 2 | 6 | 16 |

**Supplementary Table III.** Inclusion and exclusion criteria **tail bleeding**.

## Supplementary Table IV

| Experimental group | Included mice | Excluded mice- died during observation period | Excluded mice- mean tail bleeding volume±>2*SD | Excluded mice due to surgical error during MCAO surgery | Total mice |
| --- | --- | --- | --- | --- | --- |
| MCAO+tPA | 7 | 4 | 1 | 0 | 12 |
| ASA+CPG+MCAO+tPA | 11 | 2 | 1 | 2 | 16 |

**Supplementary Table IV.** Inclusion and exclusion criteria **HT quantification**.

# Supplementary Literature

1. Zheng Y, Lieschke F, Schaefer JH, Wang X, Foerch C, van Leyen K. Dual Antiplatelet Therapy Increases Hemorrhagic Transformation Following Thrombolytic Treatment in Experimental Stroke. *Stroke*. 2019;0(0):STROKEAHA.119.027359. doi:10.1161/STROKEAHA.119.027359

2. Von Kummer R, Broderick JP, Campbell BCV, et al. The heidelberg bleeding classification: Classification of bleeding events after ischemic stroke and reperfusion therapy. *Stroke*. 2015;46(10):2981-2986. doi:10.1161/STROKEAHA.115.010049

3. Yaghi S, Willey JZ, Cucchiara B, et al. Treatment and Outcome of Hemorrhagic Transformation After Intravenous Alteplase in Acute Ischemic Stroke: A Scientific Statement for Healthcare Professionals From the American Heart Association/American Stroke Association. *Stroke*. 2017;48(12):e343-e361. doi:10.1161/STR.0000000000000152

4. García-Yébenes I, Sobrado M, Zarruk JG, et al. A mouse model of hemorrhagic transformation by delayed tissue plasminogen activator administration after in situ thromboembolic stroke. *Stroke*. 2011;42(1):196-203. doi:10.1161/STROKEAHA.110.600452

5. Copin J-C, Gasche Y. Effect of the duration of middle cerebral artery occlusion on the risk of hemorrhagic transformation after tissue plasminogen activator injection in rats. *Brain Res*. 2008;1243:161-166. doi:https://doi.org/10.1016/j.brainres.2008.09.025
